# Unveiling invasive insect threats to plant biodiversity: Leveraging eDNA metabarcoding and saturated salt trap solutions for biosurveillance

**DOI:** 10.1371/journal.pone.0290036

**Published:** 2023-08-11

**Authors:** Yoamel Milián-García, Cassandre Pyne, Kate Lindsay, Adriana Romero, Robert H. Hanner

**Affiliations:** 1 Department of Integrative Biology, University of Guelph, Guelph, Ontario, Canada; 2 Ecological and Regulatory (Ecoreg) Solutions Inc., Guelph, Guelph, Ontario, Canada; University of Helsinki: Helsingin Yliopisto, FINLAND

## Abstract

The negative global impacts of invasive alien species (IAS) on biodiversity are second only to habitat loss. eDNA metabarcoding allows for a faster and more comprehensive evaluation of community species composition, with a higher taxonomic resolution and less taxonomic expertise required than traditional morphological-based biosurveillance. These advantages have positioned eDNA metabarcoding as the standard method for molecular-based detection of invasive alien species, where fast and accurate detectability allows prompt responses to mitigate their adverse effects. Here, eDNA metabarcoding is used for biosurveillance of invasive alien species regulated by Canada in high-risk areas with four main objectives: i) validate the effectiveness of eDNA metabarcoding of salt trap solutions as a molecular technique for IAS detection, ii) compare detection from DNA extracts obtained from filter quarters versus whole filters, iii) benchmark two different bioinformatic pipelines (MetaWorks and mBRAVE), and iv) compare canopy and ground level trapping. eDNA from up to five IAS (*Agrilus planipennis*, *Daktulosphaira vitifoliae*, *Lymantria dispar*, *Popillia japonica*, and *Trichoferus campestris*) were successfully detected across years from 2017 to 2022 in southern Ontario, Canada, with successful morphological validation for all except *Lymantria dispar* and *Trichoferus campestris*. Analysis of filter quarters in contrast to whole filters was demonstrated to be insufficient for effective IAS detection in each sample. All IAS were detected in only one filter quarter, suggesting a patchy eDNA distribution on the filter. The MetaWorks and mBRAVE bioinformatics pipelines proved effective in identifying IAS, with MetaWorks yielding a higher success rate when comparing molecular and morphological identifications. Ground-level and canopy-level sampling showed differential IAS recovery rates based on the molecular detection, which also varied per collection year, with all found IAS detected at the canopy level in 2022 while only one (*Lymantria dispar*) in 2020. The present study ratifies the efficacy and importance of eDNA-based detection in a regulatory context and the utility of adding eDNA metabarcoding of saturated salt trap solutions, a critical tool for IAS detection.

## Introduction

The negative effects of invasive alien species (IAS) are among the top human-mediated drivers of biodiversity loss [1]. Environmental DNA (eDNA) metabarcoding has become the method of choice for molecular-based biosurveillance [2, 3]. It allows for cost-effective, multi-species identification in diverse communities by sampling the DNA shed by the organisms into their environment [4]. Compared to conventional morphology-based identification methods, eDNA metabarcoding can reduce the time spent on species identification, sample sorting and processing, and increase the target taxa’s scope by utilizing High-Throughput Sequencing (HTS) technologies coupled with DNA reference databases. The existence of DNA reference databases (e.g., The Barcode of Life Data Systems [BOLD] [5]), which links genetic and morphological information, reduces the necessity of taxonomic expertise by assigning known identities to unclassified organisms based on molecular data.

eDNA metabarcoding has the capability to detect rare or elusive species from complex environmental samples, including species that may be considered pests, vectors of diseases, or species of conservation concern [6]. Additionally, eDNA metabarcoding causes less ecosystem disturbance than traditional biosurveillance methods and can potentially maintain the morphological integrity of detected organisms [7, 8]. The non-destructive capacity of eDNA sampling is critical in a regulatory context; it allows for specimens to be preserved for secondary morphological confirmation, which is crucial for the validation of eDNA-methods through comparison to morphology-based identification biosurveillance. Additionally, taxonomic assessment of immature stages of most arthropods is challenging; characters for identification are often dependent on adult morphology and reproductive structures [9]. DNA-based molecular identification tools such as eDNA metabarcoding provide a valuable resource for rapidly and accurately identifying morphologically indistinct IAS [10], as eDNA-based classification is independent of morphological integrity and remains invariable regardless of organism’s life stage.

Biological invasions are responsible for tremendous global impacts, including substantial biodiversity and economic losses, and management expenditures [11]. Invasive insects cause long-term environmental effects, including reduced ecosystem stability and loss of native species [12]. Invasive alien species (IAS) can destroy about 400,000 ha of forest annually in Canada [13] and are considered second only to wildfires regarding adverse effects on forests. Previous research has shown that invasions of insects alone cost a minimum of US$70.0 billion per year globally [11].

In Canada, the government is committed to strengthening the regulatory framework to control and prevent IAS. The Canadian Food Inspection Agency (CFIA) is primarily responsible for the biosurveillance of terrestrial IAS [14]. Canada has at least 272 federally terrestrial regulated species, including all the potentially invasive species, where the vast majority are insects [N = 83] (https://inspection.canada.ca/plant-health/invasive-species/regulated-pests/eng/1363317115207/1363317187811). Two (*Agrilus planipennis* and *Lymantria dispar*) out of those 83 insects are among the top IAS that cost the province of Ontario the most money [15]. Within this framework, IAS DNA-based detection, and the need to integrate molecular-based technology into regulatory policy, are continuously being explored to establish cost-effective protocols for the molecular biosurveillance [4, 16]. The main limitation for faster and more routine integration of DNA-based surveillance into current IAS biosurveillance toolkits has been linked to the lack of a thorough technological validation [17]. Four main validation categories have been recognized to support the efficacy of eDNA-based methods: experimental validation, comparative morphological/ecological validation, bioinformatic validation, and statistical validation [17]. In the present case study, we aim to address three of these categories. The first is to validate eDNA findings by comparing results from filter quarters versus whole filters and exploring whether the former resembles the same detection rate as the latter (experimental validation). Second, to compare molecular-based detection to traditional morphological-based classification (comparative morphological/ecological validation). The third is to compare outputs generated from two informatics pipelines for DNA data processing (bioinformatic validation). Validation of bioinformatics pipelines can be daunting when considering the plethora of options available for DNA metabarcoding data analyses [18]. Among other things, bioinformatic pipeline selection is linked to the users’ needs and informatics expertise [18]. In a CFIA regulatory context, user-friendly pipelines can support end-user eDNA validation by officially recognizing its utility and acceptable standards for case-specific studies such as IAS detection. In this context, we chose two bioinformatic pipelines (MetaWorks [19] and mBRAVE [https://www.mbrave.net/] [20]) out of the more than thirty-one available [18]. The Multiplex Barcode Research and Visualization Environment (mBRAVE) is a multi-user and friendly platform that supports storing, validating, and analyzing metabarcoding data. This free server-based system is built on the BOLD platform and supports rapid species identification without demanding a specific user’s informatics infrastructure or expertise. It also offers a secure collaboration and sharing environment [21], which can be essential for end-user eDNA validity recognition, especially for industry collaborators and regulatory agencies. On the other hand, MetaWorks is a free and open-source software for scalable metabarcoding data analyses of the most popular DNA barcodes [19]. It uses a naive Bayesian classifier and allows sequence composition-based taxonomic assignments [19] with associated confidence measures (bootstrap support values) [19, 22] but also requires a basic bioinformatics background and infrastructure.

In the present study, eDNA metabarcoding was used for the molecular biosurveillance of Canadian-regulated terrestrial IAS in high-risk areas with four main objectives: i) validate the effectiveness of eDNA metabarcoding as a molecular technique for IAS detection during a multi-year sampling effort conducted by the CFIA within a regulatory context, ii) compare the capacity of IAS detection when using DNA extracts obtained from filter quarters versus whole filters; iii) benchmark two different bioinformatic pipelines for metabarcoding data analysis (MetaWorks [19] and mBRAVE [https://www.mbrave.net/] [20]) within the context of IAS detection; and iv) compare the capacity of IAS detection when sampling at the canopy and ground levels. To reach these goals, the present study employs an eDNA metabarcoding approach on saturated salt trap solutions (collection fluid) collected from Lindgren funnel traps and focuses on detecting IAS regulated by Canada, adopting a method similar to the one used in previous studies [4, 23].

## Materials and methods

### Target species

The CFIA monitors species considered both invasive and regulated pests. The target species for the current study included all invasive insects regulated by the CFIA (Accessed on May 25, 2022). In addition to invasive species, the list of pests regulated by the CFIA includes 29 families and 64 genera of insects, five families and seven genera of bacteria, 22 families and 31 genera of fungi and 13 families and 23 genera of plants. For simplicity, we here refer to both CFIA-regulated invasive species and regulated pests as “invasive alien species” (IAS). The present study targeted wood-boring beetles considered as IAS, including long-horned beetles (Cerambycidae) (e.g., *Anoplophora glabripennis*, *Anoplophora chinensis*, *Tetropium fuscum*, *Tetropium castaneum*, and *Aromia bungii*). Additional species were added to the regulated species list based on noted exceptions in the CFIA list. For example, the CFIA-regulated species list states that *Phytophthora* species are regulated except for a listed subset. Therefore, the additional regulated but not listed species were added (see S1 Table).

### Collection locations

Lindgren funnel traps were deployed at four undisclosed locations in southern Ontario, Canada, in line with the CFIA’s 2019 regulatory survey program aimed at detecting insects introduced to Canada through high-risk pathways. The selected locations were Oshawa, Chatham-Kent, Brantford, and Brampton. For each location, six traps were strategically placed at six different sites near tree species known to be hosts of the target invasive alien species (IAS). This process was repeated twice, resulting in a total of 48 field samples collected in 2019. In the subsequent year, Lindgren funnel traps were set up in two additional southern Ontario locations: Greater Toronto Area and Hamilton. Similarly, six traps were placed at two sites on two different dates, yielding 24 samples. Furthermore, three more locations (Peterborough, Windsor, and Fort Erie) were sampled twice in 2022, resulting in 30 additional samples. To enhance the analysis, datasets from traps collected in 2017 and 2018 were also included [4, 23]. S1 Fig. contains an interactive map displaying the distribution of all the collected sites from 2017 to 2022. Please refer to the supporting material for further details.

### Collection protocol

Lindgren funnel traps were set up with Ultra High Release (UHR) Ethanol Lures from Synergy Semiochemical Corp, including fuscomol and fuscomol acetate as baits. To preserve trapped insect specimens and samples for eDNA analysis, the collection cups were filled with a saturated saltwater solution (2 kg of NaCl per 5 L of water). These traps were deployed at sampling sites following the CFIA’s Survey Protocol: Invasive Alien Species Forestry Trapping (2019) during the summer of 2019 and 2020. Specimens caught in the traps were removed from the solution and sent to the CFIA’s entomology laboratory. The saturated salt trap solutions were transferred to new Whirl-Pak bags and shipped to the Hanner Laboratory at the University of Guelph, Ontario, Canada. They were temporarily stored at -80°C until further processing in the laboratory.

Unfortunately, the collection fluid from two traps (trap 2 and trap 5) placed on August 26^th^, 2019, in Brampton could not be successfully collected. Additionally, the sample collection bag for trap number 3, collected on August 16^th^, 2020, in Hamilton, was empty as the salt trap solution had completely evaporated at the sampling site. Insects collected during the 2019–2020 collections were not retained, while those from the 2022 collection were sent to the Hanner lab for morphological validation.

### Filtration

The samples were taken out from the -80°C freezer and transferred to decontaminated beakers, allowing them to thaw at room temperature overnight. All filtration equipment was sterilized using a 50% bleach solution or ELIMINase from Decon Labs, Inc., followed by thoroughly rinsing with deionized water. Before filtration, the samples were inverted to resuspend any settled particles within the bag. Filtration of salt solutions was carried out using Nitrocellulose Mixed Ester membrane filters (47 mm diameter, 1 μm pore size) from Sterlitech. The filtration setup consisted of a three-piece manifold connected to a GAST vacuum pump from GAST Manufactured, Inc, utilizing magnetic filtration cups from Pall. The filter cups were covered with Kimwipes throughout the procedure to prevent DNA contamination. Some samples required multiple membranes due to the presence of a high amount of debris, leading to filter clogging. The filtration time for each sample and the number of filters used were carefully documented. As a negative control, one sample of a saturated salt solution prepared in the laboratory underwent filtration as well. The filters were then placed in sterile Petri dishes and stored at -80°C until further processing for eDNA extraction.

### eDNA extraction

Following the described steps, we performed eDNA extraction on the total number of filter membranes resulting from filtration, including negative controls. The DNeasy Blood and Tissue Kit from Qiagen was used with modifications to the manufacturer’s protocol [24]. Each filter was thawed and divided into quarters, then further cut into strips using sterile razor blades. Small strips from each quarter were placed in individual 2 mL microcentrifuge tubes along with approximately 250 mg of glass beads (0.75–1 mm diameter). Buffer ATL (380 μL) was added to each tube, and the tubes were subjected to disruption in a Qiagen TissueLyser II for 1 minute at 30 Hz. After spinning down the samples, Proteinase K (20 μL) was added to each tube, followed by vortexing and overnight incubation at 56°C at 700 rpm on the orbital shaker. The tubes were vortexed again, spun down, and Buffer AL (400 μL) was added. After vortexing and incubating at 56°C for 10 minutes, ethanol (96–100%) (400 μL) was added and vortexed for another 30 seconds. All samples, including any residues, were transferred to DNeasy Mini spin columns and centrifuged at 11,000 g for one minute. The columns were then washed twice using AW1 and AW2 buffers, following the manufacturer’s protocol. After each wash, samples were centrifuged at 11,000 g for one minute after the first wash, and then centrifuged at 17,000 g for 5 minutes after the second wash. Flow-through and collection tubes were discarded, and the DNeasy Mini spin columns were placed into clean 1.5 mL LoBind microcentrifuge tubes (Eppendorf). Buffer AE (100 μL) pre-warmed at 70°C was added onto the DNeasy membranes, incubated at room temperature for 15 minutes, and finally centrifuged for elution at 11,000 g for five minutes.

For the 2019 and 2022 CFIA collections, eDNA extracts from each quarter of the same trap were pooled into a single tube. In contrast, for the 2020 CFIA collection, extracts from each quarter were kept separate. In the latter case, 20 μL of eDNA extract from each quarter belonging to the same trap was transferred into a single fifth tube, allowing the evaluation of whether a filter fragment represents the species diversity of the whole filter. Consequently, we kept and analyzed both pooled and unpooled eDNA for each filter of the 2020 collection. If this approach enables the effective detection of invasive alien species (IAS), using only a filter fragment in each sample might be enough moving forward, decreasing sample processing costs and time, while the remaining filter material can be stored as a backup for further analysis or validation as needed.

The DNA concentrations of the eDNA extracts were determined using a Qubit 4.0 fluorometer with the Qubit dsDNA High Sensitivity kit (Thermo Fisher Scientific), using 3 uL of the DNA extracts and measuring ng/uL of DNA three times (ten minutes apart) for each sample. Qubit quantifications provide a fluorescent-based estimation of DNA concentration while comparing the intensity of fluorescent dye bound to target dsDNA, in this case, to dye binding standards (DNA probes) of known concentration. As there is a dynamic in the dye intercalation and, consequently, variation over time in the physical interaction between the dye and the target dsDNA, estimations of DNA concentrations were taken three times and ten minutes apart. Values from each estimation were averaged, and standard deviations were calculated to increase DNA concentration estimation accuracy and evaluate dispersion from the mean values. A 5 μL subsample from each extract was also visually assessed for eDNA presence and quality using 1% agarose gel electrophoresis. All eDNA extracts were stored at either 4°C for immediate use or -20°C for short-term storage before library preparation for High-Throughput Sequencing (HTS).

### Library preparation

The COI gene high-throughput sequencing library was prepared through a two-step PCR process following the same Hanner Laboratory standardized protocols and already published [4, 25]. In the first PCR, a ~407 base-pair segment of the mitochondrial genome (mtDNA) was amplified as a single fragment for all samples. The forward primers mLepF1_MiSeq/RonMWASPdeg_MiSeq and reverse primers LepR1_MiSeq/HCO2198_MiSeq were used, along with Illumina adaptors. Each reaction included a 25 μL final volume with 2.5 μL of DNA template, 12.5 μL of 2X KAPA HiFi HotStart Ready Mix, and 0.2 μM of each primer. The cycling conditions consisted of 94°C (2 min), followed by 5 cycles of 94°C (40 s), 45°C (40 s), 72°C (1 min), then 35 cycles of 94°C (40 s), 51°C (40 s), 72°C (1 min), and a final extension at 72°C (5 min) [4]. The PCR products were then analyzed on a 1% agarose gel and purified using 0.8-1x NGS magnetic beads (Machery-Nagel) ratio according to the manufacturer’s instructions.

For the second PCR, de novo synthesized index primers equivalent to the Nextera XT Index Kit were incorporated into the COI-Illumina adaptor sequence amplicons. This PCR step introduced unique index primer combinations for each sample. Reactions were set up in a final volume of 50 μL, containing 25 μL of 2X KAPA HiFi HotStart Ready Mix, 10 μL of molecular biology grade water, 5 μL of each index primer (10 μM), and 5 μL of the first cleaned-up PCR product. The PCR profile included an initial denaturation at 95°C for 3 minutes, followed by 8 cycles of 95°C (30 s), 55°C (30 s), 72°C (30 s), and a final extension of 72°C (5 min) [4, 26]. The PCR products were visualized on a 1% agarose gel, purified with a 0.6-1x NGS magnetic beads (Machery-Nagel) ratio, following the manufacturer’s instructions.

The second PCRs were conducted in separate rooms and PCR workstations from the first PCR to minimize contamination. DNA extracts and post-PCR products were treated and processed in different areas, following standard protocols for eDNA library preparation in the Hanner laboratory.

### High-Throughput sequencing

The Genomics Facility of the Advanced Analysis Centre (AAC) at the University of Guelph conducted the sequencing process. To prepare the sequencing libraries, the SequalPrep Normalization Kit from Thermo Fisher Scientific was used for normalization. The libraries were then pooled and quantified with the Qubit dsDNA HS assay kit (Thermo Fisher Scientific), while fragment size was checked using a Bioanalyzer HS DNA Chip from Agilent. Only libraries that passed the quality control were selected for sequencing. An Illumina MiSeq System with a MiSeq reagent kit v3 (600 cycles) was employed for sequencing, with each sample limited to 1% of the run (with a maximum of 100 samples per MiSeq run). The MiSeq Reporter software was utilized for demultiplexing and adapter trimming, resulting in two paired-end raw FASTQ files per sample.

### Data analysis

The quality control process for each raw data file (FASTQ files) involved assessing base sequence quality scores, per base sequence content, sequence length distribution, sequence duplication levels, overrepresented sequences, and adapter content using FastQC (http://www.bioinformatics.babraham.ac.uk/projects/fastqc/). Summary reports for each dataset were generated using MULTIQC [27]. For COI data analysis, mBRAVE (multiplex barcode research and visualization environment) (http://www.mbrave.net/) was utilized. mBRAVE is a multi-user platform that supports the storage, validation, and visualization of High-Throughput Sequencing (HTS) data. The specific parameters set within the mBRAVE platform were the same already established in previous analyses conducted by our laboratory and proven to be effective under similar experimental conditions [4, 25]: (1) trimming (30bp from the front and end, with a resulting trim length of 450bp); (2) filtering (minimum quality value (QV) of 20qv, minimum length of 200bp, maximum bases with low QV [<20] limited to 25%, and maximum bases with ultra-low QV [<10] limited to 5%); (3) pre-clustering threshold: none, ID distance threshold: 3%, minimum OTU size: 5, and OTU threshold: 2%; (4) assembler minimum overlap of 20bp and maximum substitutions of 5bp; (5) The resulting sequences from this protocol were compared to libraries assembled from BOLD (Barcode of Life Data System [5]) records and matched to a BIN (alphanumeric code representing a molecular operational taxonomic unit) when possible. Each BIN represents a putative species and includes accompanying taxonomic information for the included records when available [28].

In addition to mBRAVE, MetaWorks was used as an alternative analysis to investigate the presence of regulated species [19]. MetaWorks is a pipeline for analyzing High-Throughput Sequencing data and is beneficial for its ability to analyze different markers and resulting statistical bootstrap confidence values. MetaWorks takes raw paired-end reads as input, pair reads, then trims primers using CUTADAPT. Following trimming, MetaWorks dereplicates, and denoises reads using VSEARCH, then clusters and assigns taxonomy. An RDP (ribosomal database classifier) v2.13 was used for taxonomic classification (https://github.com/terrimporter/CO1Classifier), accessed April 14, 2022. The parameters for MetaWorks are as follows: (1) pairing (min quality: 20; min overlap; 25, the max fraction of mismatches: 0.02; min fraction of overlap: 0.90); (2) trimming and filtering (min length: 200bp; error rate: 0.1; end quality score: 20,20; min adapter overlap: 3; max number of N’s: (3); primer trimming for previously used COI primer cocktails reported in Milián-García et al., 2020 [4]. MetaWorks outputs a results table including Exact Sequence Variants (ESVs), sequences, taxonomic assignments, and bootstrap support values for each taxonomic rank (S2 Table).

In order to determine if any regulated species were present in the samples, the outputs from mBRAVE and MetaWorks were analyzed. The species returned by mBRAVE and MetaWorks (equal to and above a bootstrap value of 0.97) were compared against the updated regulated species lists in RStudio (version 022.02.0+443). To visualize the spread of regulated species, interactive and static maps were created using the leaflet, mapview, html widgets, and webshot packages in R.

As metabarcoding reference databases often do not include all species of interest, the amount of missing data in the reference databases used by mBRAVE and MetaWorks was assessed. The regulated species list was compared against the databases used by both pipelines. Since mBRAVE uses BOLD, genus-specific data was accessed from BOLD on May 31, 2022, in R. Each genus from the regulated species list was mined from BOLD, which allowed for the regulated species to be compared to each species from BOLD. Since MetaWorks uses an RDP classifier [19] (https://github.com/terrimporter/CO1Classifier), the regulated species were compared to the training set used (accessed on May 19, 2022). The effectiveness of a pipeline for IAS detection was considered based on the absolute number of molecular detections that matched morphological conformation. The higher the number of matches, the higher the efficacy of the bioinformatic pipeline in generating validated results.

To assess potential contamination and tag-jumps, metabaR [2] was used after initial bioinformatic analysis with MetaWorks [19]. metabaR is an R package that evaluates data quality by attempting to identify molecular artifacts using taxonomic outputs from previous bioinformatic pipelines, sample information, and PCR attributes. Each sampling year was analyzed separately in metabaR using the ESVs from MetaWorks and plate diagrams to determine the location of positive and negative controls. All input files were created manually, except for the MOTUs file, which was populated using a custom script, where duplicates were removed as per metabaR instructions. Data quality was assessed using each collection year’s summary_metabarlist() and gghill_rarefaction() functions. Contaminants and tag-jumps were identified using contaslayers() and tagjumpslayer(). Additional metabaR [2] functions were utilized for data visualization.

## Results

The COI datasets analysis of 2017 and 2018 samples generated 4,105,266 and 22,798,502 reads from 12 and 48 traps, respectively (Table 1). From the complete filter analysis of the 2019 and 2020 salt trap solutions, 14,189,292 and 39,580,980 sequence reads were generated from 23 and 46 samples, respectively (Table 1), while the quarter filter analysis of the 2020 samples yielded 45,115,854 sequence reads (Table 1), including reads observed in the negative controls (e.g., PCR negative and Sequencing controls). Similarly, 16,814,552 sequence reads were obtained for the 2022 samples, including field negatives and laboratory controls. The number of exact sequence variants (ESVs) among collection years ranged from 664 to 10,583 and comprised 35 and 673 species, respectively (Table 1).

**Table 1:** The number of raw sequence reads (Seq reads) that were generated per dataset analyzed in the present study, including Exact Sequence Variants (ESVs), number of Phyla (Phylum), Orders (Order), Genera (Genus), species (Species), and invasive alien species (IAS) detected based on eDNA after bioinformatic analysis with a bootstrap cut-off of 0.97 (MW = MetaWorks, and mBRAVE).

Dataset	Seq reads	ESVs	Phylum	Order	Family	Genus	Species	IAS (MW)	IAS (mBRAVE)
2017	4,105,266	664	2	12	32	35	35	1	1
2018	22,798,502	10,583	17	67	251	557	673	3	3
2019	14,189,292	5,051	13	53	204	397	445	4	5
2020	39,580,980	4,780	13	42	180	406	466	5	4
2020 Q	45,115,854	4,177	13	44	185	404	451	5	2
2022	16,814,552	3,960	9	42	182	389	444	4	3

**Fig 1:**
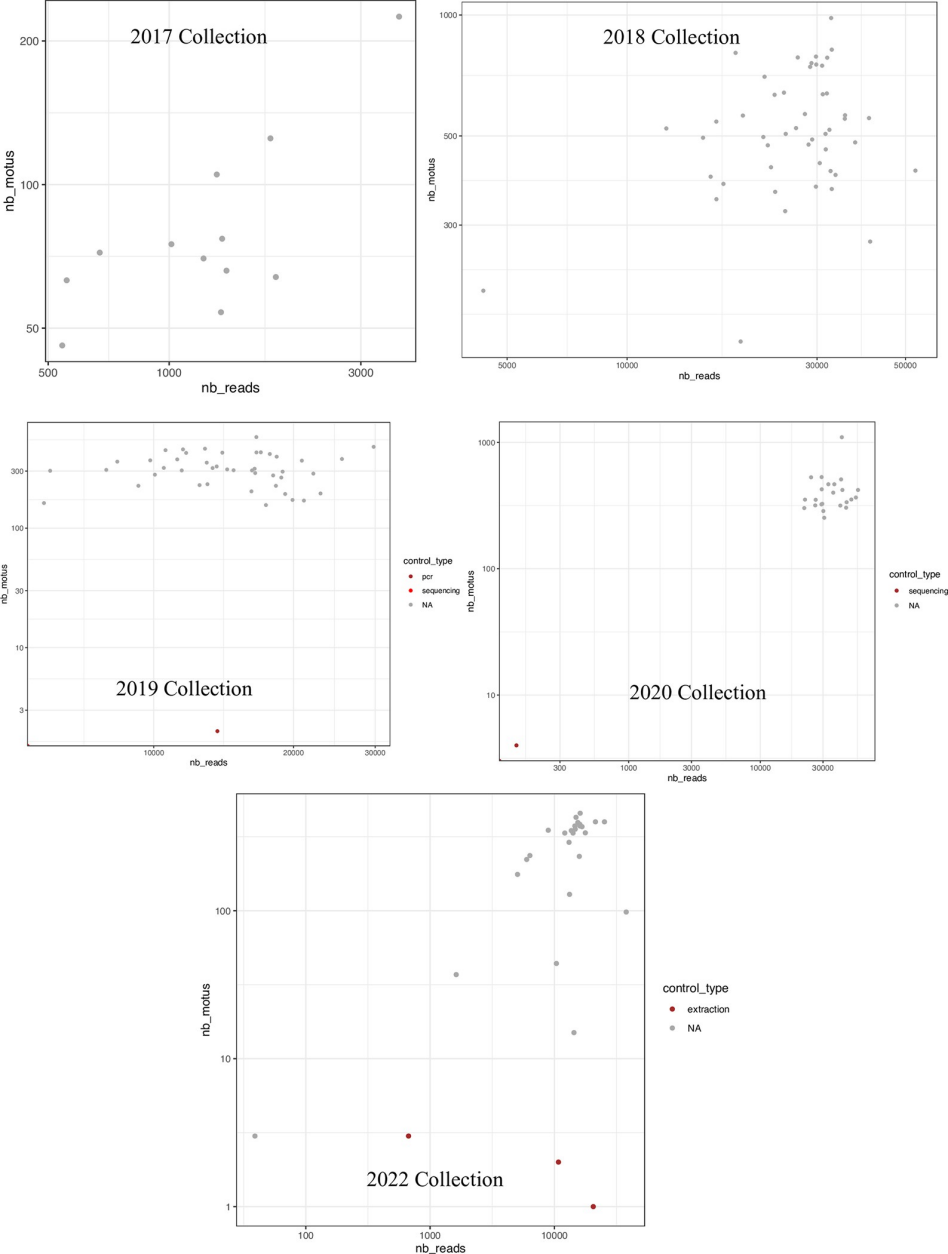
Representation of MOTU numbers (nb_motus) versus the number of reads (nb_reads) per collection. Samples (NA) are represented in grey, while sequencing and PCR controls are represented in red and brown.

**Fig 2:**
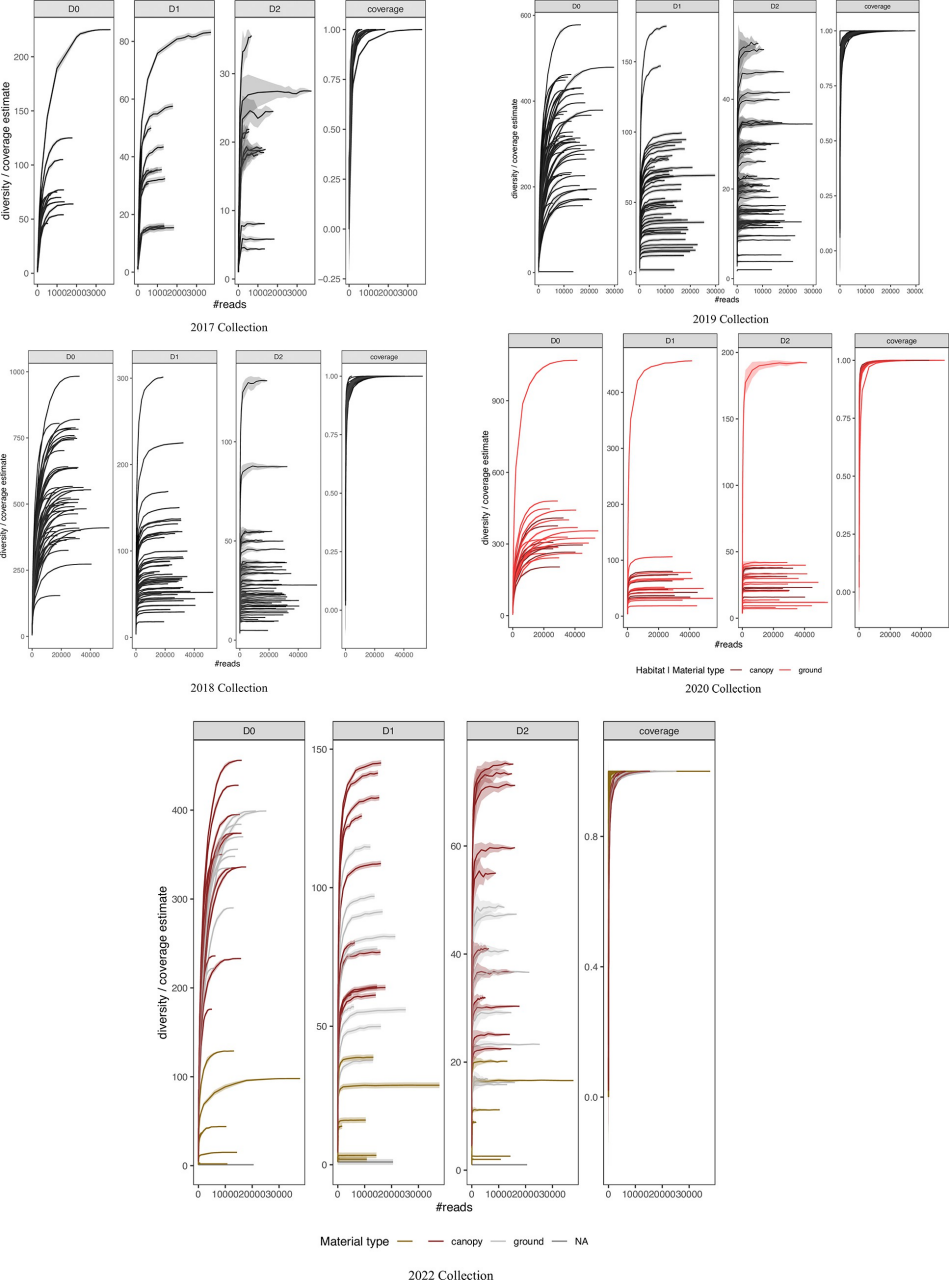
Rarefaction curves per collection year (2017 and 2018) constructed with three diversity indices. D0 is species richness, D1 approaches the exponential of the Shannon index, and D2 is inverted of the Simpson index [2, 29]. Coverage refers to the Good’s coverage index [2]. Rarefaction curves per collection year (2019 and 2020) constructed with three diversity indices. 50 is species richness, D1 approaches the exponential of the Shannon index, and D2 is inverted of the Simpson index [2, 29]. Coverage refers to the Good’s coverage index [2]. Sample types (canopy and ground) are distinguished in the 2020 collection.

**Fig 3:**
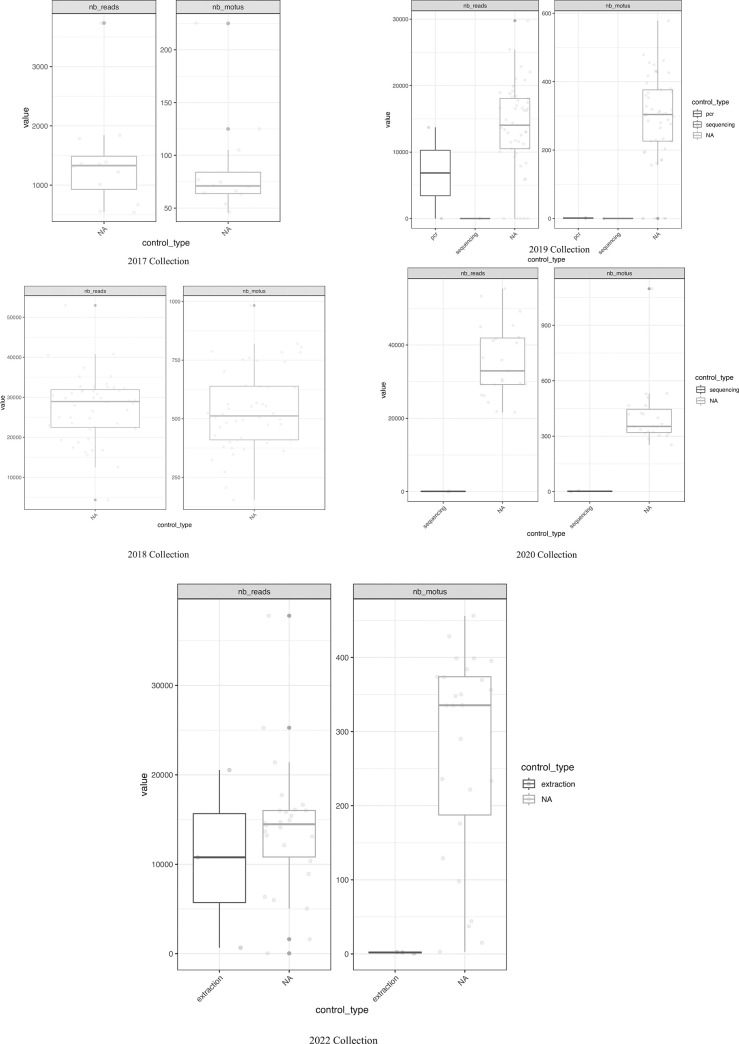
Total number of reads (nb_reads) after bioinformatic analyses and number of MOTUs (nb_motus) per collection year and sample type. NA: Represent the data generated from the samples while PCR, sequencing, and extraction indicate different control types.

No positive correlation was observed between the number of generated reads and the molecular operational taxonomic units (MOTUs) identified in all datasets except 2017, suggesting the decided sequencing depth in most cases was enough to recover the sample diversity given the primer cocktails used (Fig 1). These results were also ratified by rarefaction curves reaching the plateau for all samples in every collection year, except some in 2017 (e.g., D2; Fig 2). The sample analysis for each dataset in their experimental context showed no amplification issues during library preparations. However, it also highlighted a considerable amount of reads in PCR controls (e.g., 2019 dataset; Fig 3), although they did not represent any MOTUs (Fig 3), suggesting spurious amplifications or PCR artifacts. No inconsistencies in PCR amplification were observed for any dataset across rows or columns when analyzing the samples in their experimental context, which suggest no primer dysfunctionality or bias during library preparations. No contamination signal was observed; consequently, no contaminants were identified in the datasets based on the negative controls used.

### IAS detection and bioinformatics pipeline

**Fig 4:**
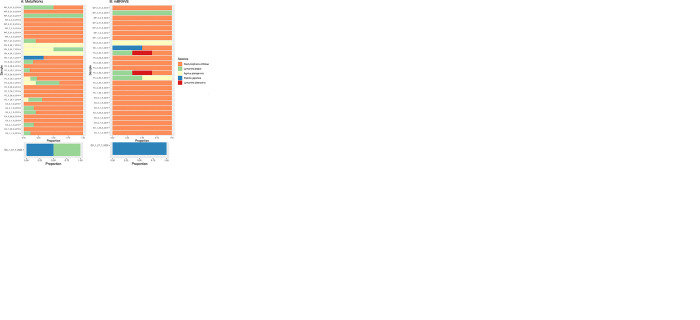
eDNA invasive alien species detection per collection year. Barplots indicate the proportion of the IAS found per sample. A: 2017 collection; B: 2018 collection; C: 2019 collection; D: 2020 collection; and E: 2022 collection.

The number of IAS detected varied between mBRAVE and MetaWorks, with the mBRAVE pipeline consistently picking up a lower number of identifications in most collections except for the 2019 collection (Table 1; Fig 4). When the number of matches between molecular and morphological detection were analyzed per bioinformatic pipeline, MetaWorks resulted in the highest number of coincidences (Fig 4; Table 2). Consequently, we focused on the species that matched both bioinformatic pipelines (all except *Rhagoletis cerasi* and *Lymantria albescens* only found by mBRAVE), considering them to be “bioinformatically validated” and used MetaWorks as our reference pipeline moving forward.

**Table 2:** Summary of IAS detections from three sites in the 2022 collection. A = Adult, L = Larval. Specimen size is presented as an average of all lengths when multiple specimens were present.

Trap	Organism(s)	eDNA detection	Morphological detection	Number of specimens	Size of the specimen(s)	Lifestage
FE_5_8_22_4	*Agrilus planipennis*	YES	YES	1	12.2 mm	A
PB_10_8–22_1	*Popillia japonica*	YES	YES	3	10.8 mm	A
	*Daktulosphaira vitifoliae*	YES	YES	2	<1 mm	L
PB_10_8_22_2	*Popillia japonica*	YES	YES	6	10.4 mm	A
	*Daktulosphaira vitifoliae*	YES	YES	2	<1 mm	L
PB_10_8_22_4	*Popillia japonica*	YES	**NO**	0	n/a	n/a
	*Daktulosphaira vitifoliae*	YES	YES	47	<1 mm	L
PB_10_8_22_6	*Daktulosphaira vitifoliae*	YES	YES	3	<1 mm	L
PB_29_8_22_1	*Popillia japonica*	YES	YES	1	11.5 mm	A
	*Daktulosphaira vitifoliae*	YES	YES	2	< 1mm	L
PB_29_8_22_3	*Popillia japonica*	YES	**NO**	0	n/a	n/a
	*Daktulosphaira vitifoliae*	YES	**NO**	0	n/a	n/a
PB_29_8_22_4	*Daktulosphaira vitifoliae*	YES	YES	2	<1 mm	A, L
	*Popillia japonica*	**NO**	YES	1	11.2 mm	A
WR_21_7_22_B1	*Daktulosphaira vitifoliae*	YES	**NO**	0	n/a	n/a
WR_21_7_22_B2	*Agrilus planipennis*	YES	**NO**	0	n/a	n/a
WR_21_7_22_B3	*Agrilus planipennis*	YES	**NO**	0	n/a	n/a
	*Daktulosphaira vitifoliae*	YES	**NO**	0	n/a	n/a
WR_21_7_22_G1	*Agrilus planipennis*	YES	**NO**	0	n/a	n/a
	*Daktulosphaira vitifoliae*	YES	**NO**	0	n/a	n/a
	*Lymantria dispar*	YES	**NO**	0	n/a	n/a
WR_21_7_22_G2	*Lymantria dispar*	YES	**NO**	0	n/a	n/a
	*Daktulosphaira vitifoliae*	YES	**NO**	0	n/a	n/a
WR_21_7_22_G3	*Lymantria dispar*	YES	**NO**	0	n/a	n/a
	*Agrilus planipennis*	YES	YES	1	12.2 mm	A
	*Daktulosphaira vitifoliae*	YES	**NO**	0	n/a	n/a

eDNA from up to five IAS regulated by Canada was found in the salt trap solutions with the highest bootstrap support (> = 0.97): *Agrilus planipennis*, *Daktulosphaira vitifoliae*, *Lymantria dispar*, *Popillia japonica*, and *Trichoferus campestris* (Table 1; Fig 4). At the time of analysis, *Trichoferus campestris* was listed as a regulated pest and is therefore treated as such in the current study, however studies of recent incursions suggest this insect is not a primary pest of trees [30] and will likely be removed from the list as a result. The analysis per collection year showed that all five IAS together were only obtained at the sites collected in 2019 and 2020. eDNA from four of these five IAS (all but *Trichoferus campestris* eDNA) was detected in the 2022 sites. All IAS detected in 2022, except *Lymantria dispar*, were successfully identified morphologically from the decanted specimens collected in that collection year. At the sample level, there were 25 eDNA-based individual detections in 2022 (Table 2), with 13 of them lacking morphological confirmation. Ten out of those thirteen detections missing morphology-based conformation came from the same collection site (Windsor). The only IAS that was genetically and morphologically confirmed to be present at Windsor was *Agrilus planipennis* (see sample WR_21_7_22_G3; Table 2). The remaining three mismatches occurred for *Popillia japonica* and *Daktulosphaira vitifoliae* and were only seen in two traps belonging to the same site (PB_10_8_22_3 and PB_10_8_22_4) where the species were also morphologically confirmed. *Lymantria dispar* and *Daktulosphaira vitifoliae* eDNA detections at Windsor in 2022 represented the only cases where the species could not be morphologically confirmed at any collected trap in the given site.

For the 2018 collected sites, eDNA from *Agrilus planipennis*, *Lymantria dispar*, *Daktulosphaira vitifoliae* was also found, while in the samples collected from sites in 2017, only eDNA from *Daktulosphaira vitifoliae* was detected (Fig 4).

### Morphological validation

In the current study, the 2022 collection was the only one where trap contents, including specimens and debris, were sent to the Hanner Laboratory for morphological validation. In 2022, four IAS species were detected, with a total of 25 individual detections over three sites, 14 traps, and four collecting dates (Table 2). Of those individual detections, 11 were confirmed by both molecular and morphological methods, one was morphological only, and the remainder (13) were molecular detections only. If assessed by site and date, there is an 89% congruence between molecular and morphological data, since IAS species not detected morphologically in one trap at a site were often detected in another trap at the same site. The Windsor site collections from July 21, 2022, show consistently low congruence between molecular and morphological identification, with only one of 11 morphologically validated detections, which suggests a sample-specific bias.

### Full filter analysis vs quarters

**Fig 5:**
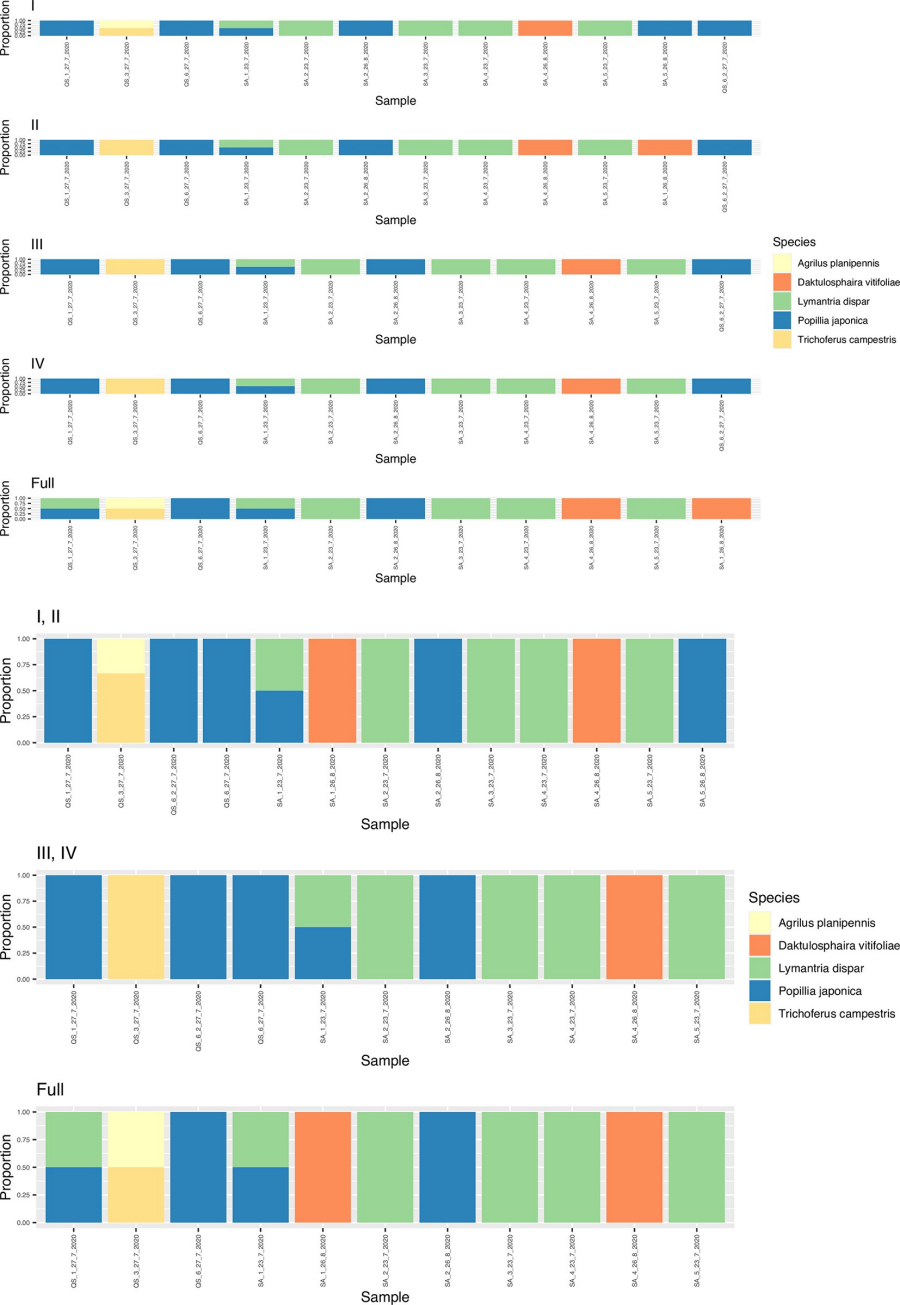
Invasive alien species (IAS) detection per 2020 collected sample. Quarter filters (I, II, III, and IV), halves (I, II and II, IV), and whole filter (full) analyses are reflected, with each colour representing one of the IAS detected based on their shed eDNA in the salt trap solution. X-axis represents individual samples and the Y-axis is the proportion of IAS found per sample.

The whole filter versus quarter analysis for the 2020 collection showed that all five IAS eDNA was detected in only one quarter (Quarter I; Fig 5). These results were consistent when comparing IAS species recovered from half and whole filters (Fig 5). *Agrilus planipennis* eDNA was missing in three out of the four quarters belonging to the same filters (Fig 5).

**Fig 6:**
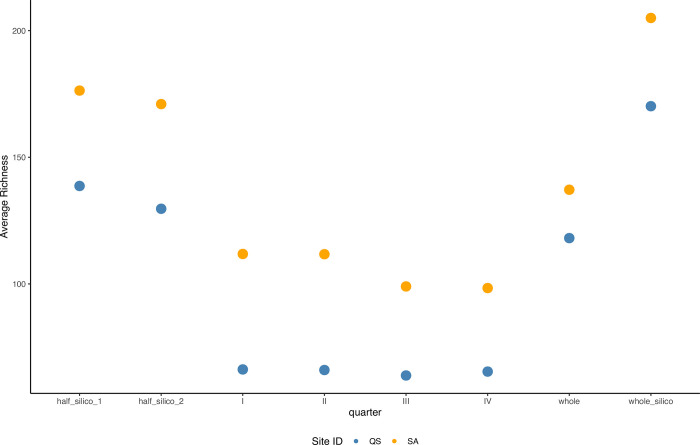
Average species richness per 2020 sample collected, including whole and quarter filter analysis. QS and SA refer to specific 2020 sampling sites. Whole and half in silico refer to the ESV bioinformatic combinations for the entire filter and their halves from filter quarters.

Additionally, species composition analysis demonstrated that quarters or halves alone did not necessarily reflect the species composition of the whole filter, with complete filter analysis consistently showing higher average species richness for all 2020 sampled sites (Fig 6).

### Canopy level sampling vs ground level sampling

**Fig 7:**
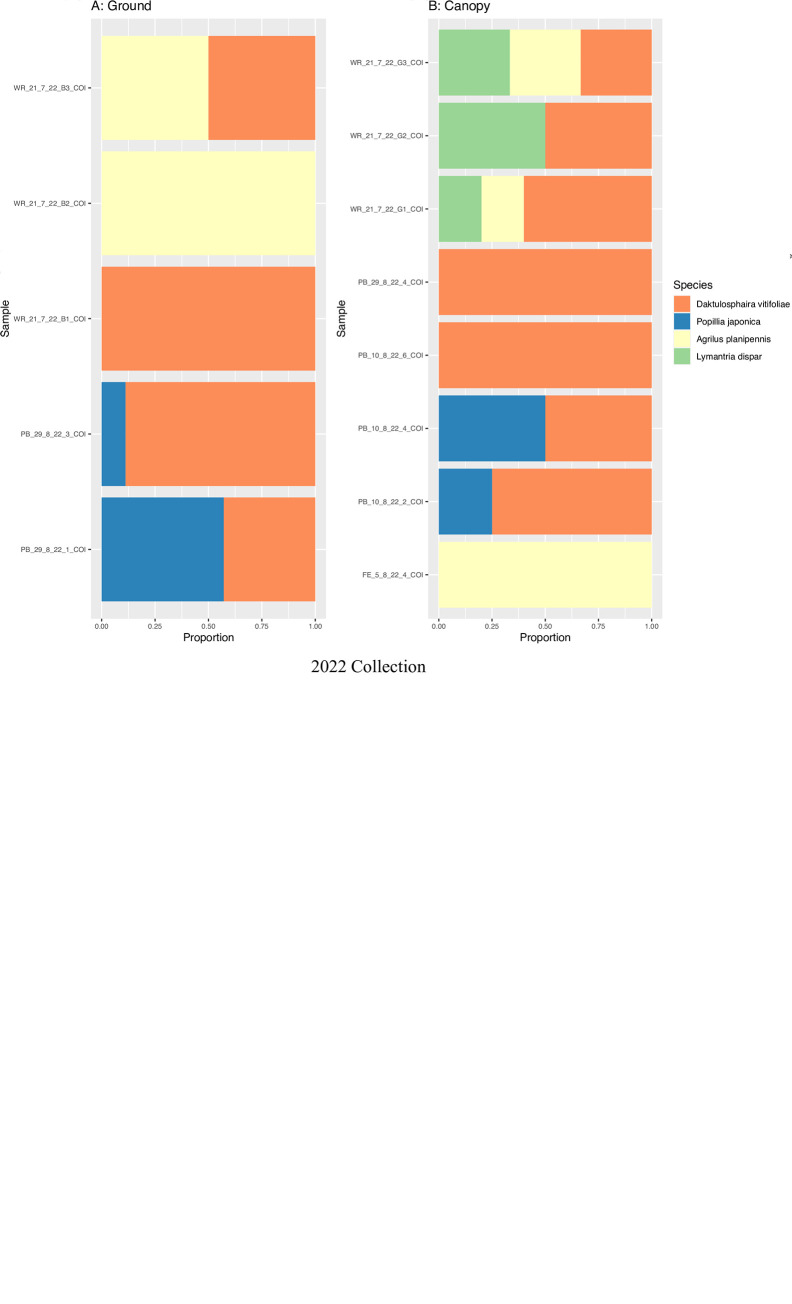
Invasive alien species detection at the ground level versus canopy level (lower panel) in the 2020 and 2022 collections.

Only eDNA from one IAS (*Lymantria dispar*) was detected at the canopy level, contrary to the five IAS that were seen at the ground sampling level during the whole filter analysis of the 2020 collection (Fig 7). On the contrary, eDNA from all four IAS detected in 2022 were recovered from samples taken at the canopy level, and only three IAS were identified during the ground-level sampling effort that year (Fig 7).

## Discussion

Due to their non-destructive and cost-effective nature, environmental DNA-based approaches, such as eDNA metabarcoding, have become the standard molecular method for multispecies identification. However, the routine implementation of eDNA methods in a regulatory context requires thorough validation to be broadly accepted by regulators [17]. In the present study, we pursue eDNA validation for IAS detection on CFIA-sampled salt trap collection fluids using a molecular multi-species identification approach and an experimental, morphological, and bioinformatic validation [17].

### Effectiveness of eDNA metabarcoding for IAS detection

eDNA from up to five invasive alien species regulated by Canada (*Agrilus planipennis*, *Daktulosphaira vitifoliae*, *Lymantria dispar*, *Popillia japonica*, and *Trichoferus campestris*) was consistently found across five years (2017–2022) in high-risk areas sampled by the CFIA.

The emerald ash borer (EAB), an invasive beetle from Asia, has caused extensive damage since its introduction to North America in 2002 [31]. The larvae of this wood-boring beetle feed on ash trees, forming tunnels under the bark and ultimately killing the trees within a few years [32]. This has led to a significant loss of ash trees, decreased biodiversity, and increased vulnerability to other invasive plants [33–35]. The economic losses from EAB are in the billions of dollars [36], making its regulation crucial [37]. In contrast, grape phylloxera, a native North American pest, exclusively feeds on grapevines and has historically caused significant vineyard losses [38, 39]. However, grafting European grapevines onto resistant rootstocks has helped manage this pest [39]. Despite being minor in Ontario, grape phylloxera remains economically significant worldwide [38, 39], emphasizing the need for early detection and population regulation. The spongy moth, introduced to Ontario in 1969 [40], defoliates various hardwood and softwood trees during outbreaks, reducing tree health and making them more susceptible to other threats. This moth is one of the most economically damaging insect pests [11], with outbreaks occurring every 7 to 10 years [40]. Spongy moths can also cause acute contact dermatitis when humans come in contact with larvae, which has implications for human health [41]. On the other hand, the Japanese beetle, native to Japan and introduced to North America in 1916 [42], then in Canada in 1939 [43], is highly destructive to turfgrass, landscape plants, and pastures. Its larvae damage grassroots, while adults heavily feed on leaves, impacting over 300 plant species in 79 families [44] and having economic impacts on over 100 of those species [42]. The Japanese beetle causes significant economic losses and requires substantial control efforts [45]. Finally, *Trichoferus campestris*, a velvet longhorned beetle native to Asia, was detected in Canada in 2002 [46]. *T*. *campestris* is native to Asia and feeds on healthy or mildly stressed adults in over 40 genera of broadleaf and conifer trees in its native range; however, it prefers apple and mulberry trees [47, 48]. While it was initially considered a primary pest of trees, recent studies suggest otherwise, indicating that it does not cause significant damage [30].

The above species detections highlight the utility of molecular biosurveillance to detect and prevent further spread. As in previous cases, an early warning for prompt actions can profoundly impact preserving biodiversity, significantly reducing economic losses and contributing to Canadians’ well-being.

### Filter quarters vs. whole filter analysis

The lack of consistent IAS eDNA detection on most filter quarters from the 2020 collection suggests a patchy distribution of eDNA on the filters. This can impact species detectability and constitute a sampling bias when using filter fragments alone for eDNA detection. Therefore, pooling extracts from all filter quarters belonging to the same sample before library preparation [4, 49] or whole filter analysis is suggested for more accurate IAS detection and the most comprehensive inference of species diversity in a sampling site. Multiple biological replicates using whole filters is desirable in contrast to using filter fragments as a backup plan or for use as voucher samples for future analysis. Present species composition analysis beyond IAS ratified that quarters or halves alone did not necessarily reflect the species diversity of the whole filter. The latter might directly affect any molecular-based inference of species presence based on filter quarters, which can be common in the eDNA field [50–52].

It is important to consider that library preparation and High-Throughput Sequencing of a given sample is a sampling process itself [2]. Consequently, sequencing results mainly reflect the diversity in the processed sample under the experimental conditions rather than the site diversity. To get a more accurate representation of site diversity, the sampling effort must be comprehensive enough, there should be no primer biases or taxonomic blind spots, and all prospective species occupying the study area should be properly documented in the reference DNA databases. Failure to meet the mentioned conditions can hinder the capacity for effective eDNA IAS identification in addition to the sampling biases stated above.

### Experimental, bioinformatic and morphological/ecological validation

The five IAS referred to above and detected across years (2017–2022) were successfully confirmed by two independent bioinformatic pipelines (MetaWokrs and mBRAVE), increasing the accuracy of eDNA-based detections and reducing the probability of false identifications. In addition, all IAS detected in 2022, except *Lymantria dispar*, were successfully identified morphologically among the specimens sampled in that collection year, confirming species presence in the sampling area. However, not every eDNA detection at a given sample was validated with morphological confirmation. Several factors including insect biomass, time in trap fluid, and environmental conditions, can affect the sensitivity of eDNA detection methods. The amount of eDNA shed into collection fluid is primarily linked to the number of specimens trapped (e.g., biomass) and the time the specimens remained in the fluid. Additional environmental conditions such as temperature and UV exposure can also influence the amount of eDNA shed by certain IAS, and its subsequent degradation. The rate between eDNA shedding and degradation directly affects the molecular detectability of a species; low rates of shedding/high rates of degradation can lead to specimens being detected morphologically but lacking eDNA identification, as seen in one case here (Trap PB_29_8_22_4). Conversely, there may be cases where a species is detected molecularly but not morphologically. This can be caused by a number of reasons including: an organism escaping a trap after shedding significant amounts of DNA, an organism’s feces, eggs or other eDNA sources falling into the trap without the organism itself, or a soft-bodied organism decays while in the trap fluid. To increase IAS detectability and to have comprehensive sampling, the present study deployed up to six traps per collection site. However, no level of replication or sampling effort can ensure 100% agreement between morphological and molecular identification in the above-mentioned scenarios. These disagreements should be considered carefully on a sample-by-sample basis, as many factors are at play, including those mentioned above and others, such as sampling bias and taxonomic expertise, which may influence results.

To further validate the eDNA-metabarcoding approach of salt trap solutions for effectively detecting IAS, morphological vouchers can be compared to molecular-based identifications (comparative/ecological validation) in rigorously prepared mock communities resembling complex environmental samples. Choosing eDNA-based detection and/or traditional morphological observations for IAS detection should be carefully considered in light of each approach’s known pros and cons. Molecular identifications offer a faster and more sensitive method than traditional approaches, which is critical in the regulatory context. Faster detection allows for detectability before species abundance reaches a point where their damaging effects are difficult to manage. Normally, when IAS are detected via traditional observations, their populations have already grown too large, which makes effective management more difficult and costly. In the 2022 collection, there were 25 individual detections with 11 of those ratified by both molecular and morphological approaches; one detection was morphological only and the remaining 13 were molecular only. The case of morphological detection only was represented by a single specimen, which might have been trapped towards the last moment of collection and may have not shed enough genetic material into the trap fluid before it was removed for morphological identification. Of the 13 detections that were molecular detection only, six of them represented cases where the species was validated in the same collection site but from another trap in that same site. If matching traditional morphological identification to molecular detection is considered the gold standard for validating eDNA results, the current success rate combining both scenarios is 68% (17 out of 25). However, as discussed above, there are well-known scenarios where molecular and morphological detections would not allow for 100% agreement, emphasizing the need to carefully evaluate molecular tools in regular biomonitoring and biosurveillance programs on a sample-by-sample basis. At the bioinformatic level, MetaWorks [19] resulted in more coincidences between molecular and morphological identifications than mBRAVE [20], although the number of IAS detected when using both bioinformatic pipelines was comparable. Altogether, it demonstrates the impact of the data analysis strategy on the final output and the need for using more than one bioinformatic pipeline for maximizing detection probability.

### Trap height

There were significant differences in IAS detectability, with ground-level traps detecting all five species in the 2020 collection, while canopy level-traps detected only one species (*Lymantria dispar*). On the contrary, most IAS detected in 2022 were found at the canopy level compared to the ground-level sampling. These differences can be partially explained through species behaviours. However, similarly to current findings, studies often find mixed results concerning trap height and should be considered on a species-by-species basis [53, 54]. Previous behavioural studies show that Japanese beetles (*Popillia japonica*) are more attracted to traps near ground level [55, 56]; this is where males often fly in search of females [44]. Some studies show Emerald ash borer (*Agrilus planipennis*) (Coleoptera, Buprestidae) prefers traps placed in mid to high canopy in contrast to those placed near the ground [57, 58]; this corresponds with previous findings [59] that observed most adult flights of emerald ash borer occurring in the upper and mid-canopy region. Studies on other Buprestid and Cerambycid species have mixed results, with both taxa either preferring the canopy, ground, or with no preference depending on the species [53, 54, 60–62]. The specific flight behaviour of *T*. *campestris* (Coleoptera: Cerambycidae) in relation to forest canopy height is currently unknown, although the standard trap placement used is 1.5 m [63]. Grape phylloxera (*Daktulosphaira vitifoliae*) is commonly found near ground level since they emerge from the soil. In Ontario, grape phylloxera is suggested to prefer a trap height of 1.3 m in contrast to 0.6 m [64], while another study found no significant difference between traps at 1.5, 2.1, and 2.7 m [65]. Spongy moth (*Lymantria dispar*) males are strongly attracted to vertical objects and commonly fly up and down tree trunks, where females would most likely be encountered [66]. Various studies note greater success for ground-level traps generally below 4 m [67–72]. Collins and Potts (1932) found that traps placed at 21.3 m height did not capture any males [73] while Richerson et al. (1976a) showed that males primarily search for females at the height of the highest pheromone source [74], which tends to be below 4 meters [75]. In summary, our study showed greater success for ground-level sampling at 2020 and at the canopy-level in the 2022 collection, which partially corresponds to specific insect behaviour in each case. However, other studies of similar taxa show mixed results, therefore we suggest sampling at both the ground and canopy in future studies for better species representation until more data is gathered.

## Conclusions

eDNA metabarcoding of salt trap collection fluids is a valid molecular-based tool for rapid IAS detection, allowing morphological confirmation when needed. Complete filter processing is recommended for higher IAS detection probability instead of filter fragment analysis due to potentially patched eDNA distribution on the filters. MetaWorks and mBRAVE bioinformatics pipelines effectively allowed IAS identification, but MetaWorks resulted in a higher success rate. In the context of CFIA molecular biosurveillance, ground-level and canopy-level sampling should be considered for a more comprehensive sample regardless of specific IAS, as the effectiveness of trap height continues to be species-dependent and match the behaviour of the particular IAS found.

## Supporting information

S1 Fig Interactive map displaying the distribution of all the collected sites in southern Ontario from 2017 to 2022. (HTML)

S1 Table Up-to-date species list of terrestrial regulated species by Canada. (CSV)

S2 Table a-f. MetaWorks output tables, including Exact Sequence Variants (ESVs), ESV sizes, taxonomic assignments, and bootstrap support values for each taxonomic rank per dataset analyzed in the present study (2017–2019, 2020 quarters and full, and 2022).
(ZIP)
